# Aortoduodenal fistula and abdominal aortic aneurysm as a complication of Brucella Aortitis managed with Insitu aortic aneurysm repair: A case report

**DOI:** 10.1002/ccr3.8269

**Published:** 2023-12-03

**Authors:** Ahmad Hosseinzadeh, Jumana Zehra, Mohammad Ali Davarpanah, Mohammad Moeini Farsani, Meghdad Ghasemi Gorji, Reza Shahriarirad

**Affiliations:** ^1^ Thoracic and Vascular Surgery Research Center Shiraz University of Medical Science Shiraz Iran; ^2^ School of Medicine Shiraz University of Medical Science Shiraz Iran; ^3^ Shiraz HIV/AIDS Research Center Shiraz University of Medical Sciences Shiraz Iran

**Keywords:** abdominal aortic aneurysm, aortoduodenal fistula, *brucella* aortitis, brucellosis, Insitu aortic repair

## Abstract

**Key Clinical Message:**

Brucella aortitis should be one of the differential diagnoses of inflammatory aortic aneurysms. In situ repair of intermittent aortoenteric fitulae and repair of infrarenal aortic aneurysm with synthetic graft can be used in clean scarred fistulae.

**Abstract:**

Arterial aneurysms are very rare complications of Brucella infection. The purpose of this case report is to document a case of abdominal aortic aneurysm and primary aorto‐duodenal fistula as a complication of Brucella infection, along with the management of brucella induced aortoenteric fistula with insitu synthetic graft. We report a 53‐year‐old man with a complaint of abdominal pain and melena. Radiological evaluation revealed an inflammatory abdominal aortic aneurysm and a primary aorto‐duodenal fistula was identified during surgery. The patient underwent laparotomy, and surgical repair of the aneurysm with a bifurcated Dacron graft, while the entry of the aorto‐duodenal fistula was closed with intra‐aortic sutures. One month later, the patient tested positive for the Wright agglutination test (1:80) and Coomb's test (1:640) for brucella, and was treated with doxycycline, rifampicin, and ciprofloxacin for brucellosis. Though rare, brucella aortitis should be considered as one of the differential diagnoses of inflammatory aortic aneurysms. In situ repair of intermittent aortoenteric fistula and repair of the infrarenal aortic aneurysm with synthetic graft could be considered in a clean scarred fistula.

## Graphical abstract

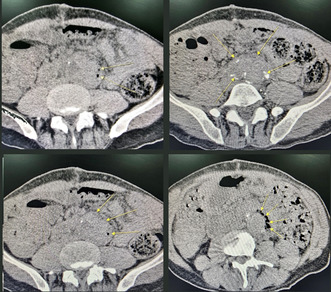

## 1 INTRODUCTION

Brucellosis is a very common zoonotic infection where the organism is transmitted to humans either through direct contact or by consuming contaminated products of the infected animals. The symptoms of brucellosis are fever, arthralgia, myalgia, headache, and weight loss. However, endocarditis, testicular abscess, and bone abscess can occur as a complication.^1^, ^2^ Arterial aneurysms such as aortic aneurysms are very rare complications of Brucella infection.^3^ However, infective endocarditis can secondarily be the cause of aneurysms in the peripheral arteries. Aortic involvement in Brucella infection is usually a mycotic aneurysm; However, pseudoaneurysms and dissecting aneurysms have also been reported.^4^, ^5^ These aneurysms later form a fistula with the surrounding structures, like aortoesophageal fistula,^6^ aortobronchial fistula,^7^ and aortoduodenal fistula.^8^

Aortoduodenal fistulas present with a classical triad of symptoms; gastrointestinal bleeding, abdominal pain, and pulsating abdominal mass.^9^ However, only 10% of the patients present with the complete triad.^10^ Mortality rates in aortoduodenal fistulas are extremely high, around 100% if untreated and almost less than 30% in patients who undergo surgical intervention.^11^ The purpose of this case report is to document the successful management of abdominal aortic aneurysm and a primary aorta‐duodenal fistula as a complication of Brucella infection and to introduce in situ aortic aneurysm repair in patients with infected aneurysms.

## 2 CASE PRESENTATION

A 53‐year‐old man came with abdominal pain and melena, along with general weakness and dizziness. He has had these symptoms intermittently during the past 4 months. His past medical history is remarkable for cardiovascular disease from the past 15 years, benign prostatic hypertrophy, and previous treatment of peptic ulcer disease. The patient had a similar episode of lower gastrointestinal bleeding 10 years ago, in which received packed cells due to a hemoglobin drop (6 mg/dL). His medications include Aspirin 80 mg once daily, clopidogrel 75 mg once daily, tamsulosin 0.4 mg once daily, metoral 25 mg once daily, and atorvastatin 80 mg at bedtime. He had no previous surgery, trauma, or any other medical condition. No history of drug, alcohol, or tobacco use. A review of systems was otherwise noncontributory.

On examination, the patient is a middle‐aged man alert, oriented to time, place, and person, without any distress. The blood pressure was 120/80 mmHg with a heart rate of 70 beats per minute and a respiratory rate of 12 on admission. The patient was afebrile. The abdomen was soft without any tenderness; however, a pulsatile mass was palpable in the supraumbilical region. Distal pedal pulses were detected. Laboratory data included a white blood cell count of 3.8 × 10^9^/L, hemoglobin concentration of 13.7 g/dL, and platelet count of 251 × 10^3^/μL. Blood urea nitrogen, creatinine, and electrolytes were normal, as were the liver function tests and coagulation tests.

**FIGURE 1:**
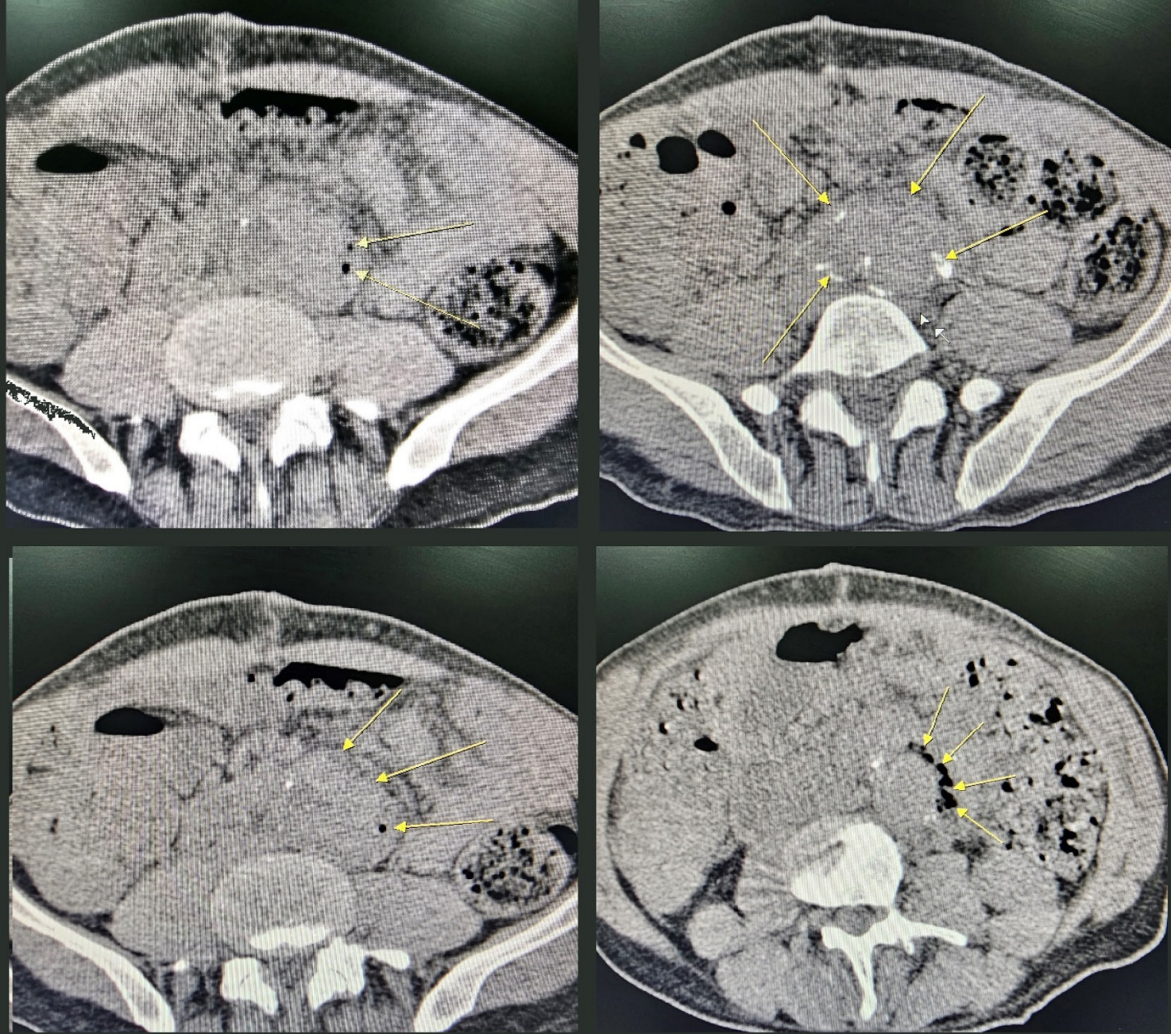
Computed tomography scan without contrast demonstrating infected aneurysm suspicious to have air bubbles in its wall. (Arrows indicate air bubbles (black spaces) and the aneurysm).

Esophagogastroduodenoscopy and colonoscopy showed no abnormalities. Spiral Computed tomography (CT) showed an infrarenal aneurysm of the length of about 11 cm and maximum anterior–posterior diameter of 7 cm and a maximum transverse diameter of 5 cm. Also, evidence of large intraluminal thrombosis at the region of aneurysmal dilation was seen. In addition, evidence of fat stranding surrounding aneurysmal dilation at the left upper quadrant of the abdomen was noted. Significant mesenteric inflammation associated with retroperitoneal inflammation was reported. Adjacent bowel loops showed wall thickening probably secondary to the extension of mesenteric inflammation. However, no signs of fistula were not reported in the CT scan (Figure 1).

Due to the short proximal neck of the aneurysm, treatment with an endovascular aortic repair (EVAR) procedure was not proposed and the patient was planned for open surgery. On exploration, severe inflammatory aortic aneurysm with multilobar adhesion with mesentery of transverse colon and duodenum was seen and after aortic clamping and aortotomy, a 2 × 3 mm fistula was discovered in the right wall of the aneurysm near the right renal artery. No duodenal dissection was performed. The fistula had no secretion or drainage and was repaired from inside of the aortic sac with prolen sutures. The patient underwent graft replacement of aortoiliac using an aorto‐bi‐iliac bifurcated Dacron graft, where end to end anastomosis was done at the infrarenal aortic neck and the common iliac arteries ends in distal. Wall of the aneurysm sac had adhesions to bowel, so debridement and resection of aneurysm wall was not possible and it was left open. The omentum was used as a flap over the prosthesis. This also prevents secondary graft infection and the formation of graft‐enteric erosion.

Postoperatively, the patient had a high Erythrocyte sedimentation rate and C‐Reactive Protein levels. The patient was treated with broad‐spectrum intravenous antibiotics for 4 weeks till C‐Reactive protein levels decreased to normal, and was discharged with oral levofloxacin and clindamycin.

**FIGURE 2:**
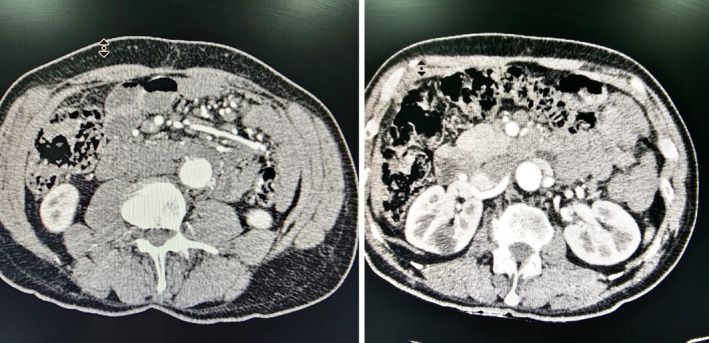
Computed tomography angiography scan demonstrating no complications 1 year after operation, with a patent in‐situ graft.

During follow‐up, the patient reported being a stockman engaged in raising livestock and consuming them. Also, he had complained of body and joint pain. On evaluation, he tested positive for the Wright agglutination test (1:80) and Coomb's test (1:640). The patient was sent to the infectious clinic where antibiotic treatment changed to doxycycline, rifampicin, and ciprofloxacin was started for 3 months. During 1 year of follow‐up, the patient reported no further complaints and complications, and CT angiography 1 year after completion of treatment was unremarkable with no complications (Figure 2).

## 3 DISCUSSION

The primary method of diagnosis of brucella is serologic testing. Brucella serology either by enzyme‐linked immunosorbent assay or agglutination is positive in almost 92% of patients with endarteritis.^12^ Agglutination tests are measured with different assays like the Wrights Agglutination test, Coomb's test, and 2‐mercaptoethanol test.^13^ Titers of 1:80 show active infection, titers of 1:160 or more with clinical presentations are highly suggestive of the brucella infection, especially in endemic areas as in our case.^14^, ^15^

For aortoduodenal fistula, esophagogastroduodenoscopy can be helpful for the diagnosis. However, the fourth part of the duodenum should also be properly evaluated.^16^ Other modalities like a CT scan of the abdomen prove to be more helpful. CT with intravenous contrast is the most informative initial test if there is a suspicion of aortoduodenal fistula in inflammatory aortic aneurysms.^17^ CT in favor of air in the aortic wall,^18^ or periaortic soft tissue, fluid accumulation, a breach in the anterior side of the aortic wall, loss of fat pad,^19^ and bowel thickening^20^ are specific signs. For aortoduodenal fistula, arteriography of the abdominal aorta has also not proven to be of high sensitivity.^16^, ^21^ So, the diagnosis should be made on the CT findings and the clinical presentation of the patient.

The recommended treatment for brucellosis by the World Health Organization (WHO) is doxycycline and streptomycin/rifampicin for 45 days.^19^ However, for the treatment of brucella arteritis, the antibiotic regimen must include two or three of these five antibiotics: fluoroquinolone, tetracycline, aminoglycoside, sulfanilamide, and rifampin for 3 months with surgical intervention.^12^ The operative approach for the revascularization of infected aneurysms is controversial.^22^ In the setting of non‐infectious patients, EVAR may prove to be more helpful. It is less invasive and more feasible. However, the traditional treatment is the in‐situ graft replacement and the extra anatomical bypass with aortic ligation.^23^ Furthermore, the decision on the method should be made by accessing the anatomy of the aneurysm, the clinical position of the patient, the degree of contamination, and the clinical experience of the surgeon.

EVAR is the preferred operation in high‐risk patients because it is minimally invasive and the ability to stabilize patients with bleeding is rapid. In unstable patients, it is recommended a bridge to definitive open repair surgery as in aortoenteric fistulas.^24^ However, the presence of a proximal aneurysmal neck or the size of the neck for fixation of an endograft in EVAR is a significant entity in ruling in/out this procedure.^18^ As in our case, due to the short proximal neck of the aneurysm in our patient, we could not opt for an EVAR as proximal aortoprosthetic anastomosis could not have been successful.

Extra‐anatomic bypass with aortic ligation is advisable in patients with extensive involvement of surrounding tissues as in widespread periaortic sepsis.^25^ In situ graft replacement with Dacron graft or polytetrafluoroethylene is performed if the adjacent contamination is less.^26^, ^27^ However, to decrease graft‐related complications like secondary fistula formation, placing an omentum between the graft and the duodenum is recommended.^28^

## 4 CONCLUSIONS

Though rare, brucella aortitis should be considered as one of the differential diagnoses of inflammatory aortic aneurysms. Moreover, if the patient presents with gastrointestinal bleeding, the primary aortoduodenal fistula needs to be taken into consideration. In situ repair of intermittent aortoenteric fistula and repair of the infrarenal aortic aneurysm with synthetic graft could be considered in a clean scarred fistula.

## AUTHOR CONTRIBUTIONS

**Ahmad Hosseinzadeh:** Conceptualization; supervision. **Jumana Zehra:** Data curation. **Meghdad Ghasemi Gorji:** Data curation. **Mohammad Moeini Farsani:** Investigation. **Mohammad Ali Davarpanah:** Methodology; supervision. **Reza Shahriarirad:** Supervision; writing – original draft; writing – review and editing.

## FUNDING INFORMATION

No financial support was received for this report.

## CONFLICT OF INTEREST STATEMENT

The authors declare that they have no competing interests.

## ETHICS STATEMENT

Written informed consent was obtained from the patients in our study. The purpose of this research was completely explained to the patients and they were assured that their information will be kept confidential by the researcher.

## CONSENT

Written informed consent was obtained from the patient for the publication of this report. A copy of the written consent is available for review by the Editor of this journal.

## Data Availability

All data regarding this study has been reported in the manuscript. Please contact the corresponding author if you are interested in any further information.
